# Functionalization of Polymer Surface with Antimicrobial Microcapsules

**DOI:** 10.3390/polym14101961

**Published:** 2022-05-11

**Authors:** Iva Rezić, Maja Somogyi Škoc, Mislav Majdak, Slaven Jurić, Katarina Sopko Stracenski, Marko Vinceković

**Affiliations:** 1 Department of Applied Chemistry, Faculty of Textile Technology, University of Zagreb, 10000 Zagreb, Croatia; mislav.majdak@ttf.hr; 2 Department of Materials, Fibers and Textile Testing, Faculty of Textile Technology, University of Zagreb, 10000 Zagreb, Croatia; maja.somogyi@ttf.hr; 3 Faculty of Agriculture, University of Zagreb, 10000 Zagreb, Croatia; sjuric@agr.hr (S.J.); ksstracenski@agr.hr (K.S.S.); mvincekovic@agr.hr (M.V.)

**Keywords:** microcapsules, dip coating, encapsulation, spectroscopy, microscopy, antibacterial silver

## Abstract

The development of antimicrobial polymers is a priority for engineers fighting microbial resistant strains. Silver ions and silver nanoparticles can assist in enhancing the antimicrobial properties of microcapsules that release such substances in time which prolongs the efficiency of antimicrobial effects. Therefore, this study aimed to functionalize different polymer surfaces with antimicrobial core/shell microcapsules. Microcapsules were made of sodium alginate in shell and filled with antimicrobial silver in their core prior to application on the surface of polymer materials by dip-coating methodology. Characterization of polymers after functionalization was performed by several spectroscopic and microscopic techniques. After the characterization of polymers before and after the functionalization, the release of the active substances was monitored in time. The obtained test results can help with the calculation on the minimal concentration of antimicrobial silver that is encapsulated to achieve the desired amounts of release over time.

## 1. Introduction

Microorganism strains that are resistant to a wide range of antibiotic-based penicillin drugs are known as the members of the “*super-bugs*” group. Conventional drugs that are often used cannot harm them, so they (e.g., methicillin, dicloxacillin, nafcillin or oxacillin) are not efficient against special strains of *Staphylococcus aureus*. The problems with antimicrobial resistance become even more important during the COVID-19 pandemic, since more people were hospitalized and suffered from compromised immune systems. The size of the nanoparticles in the antimicrobial coatings is much smaller than the size of the bacteria which is crucial for effective penetration through the cell wall and causes undesirable effects on microorganisms [1,2].

Silver is a powerful antimicrobial reagent [3,4,5,6]. During polymer surface modification, different techniques are used for characterization [7,8,9,10,11,12,13]. The most frequently used methods are different varieties of spectroscopic techniques [14,15,16,17,18]. In addition, liquid or ion chromatography helps with efficient sample preparation and the analysis of particular metal ions [19,20,21,22,23]. However, for complex samples such as encapsulated nanoparticles, the combination of different methodologies is needed.

Although the process of microencapsulation technology originates from the 1940s, during the last 10 years, encapsulated products have been extensively used in many industries, such as the agricultural, food, cosmetic and textile industries. In medical applications in particular, microencapsulated materials are showing an increase in the market since they can offer the prolonged release of the active compounds [3]. The encapsulation process is a technique in which the small particles are surrounded by the desired coating, resulting in a typical diameter of around 2 to 2000 μm. The average thickness of the walls is 0.5–150 μm and the core can contain 20 to 95% of the total weight. The choice of the methodological encapsulation technique depends on the desired purpose of the microcapsules.

There are many advantages of microencapsulation, and the most prominent are: the protection of the inner active species, controlled release, practical handling and targeted delivery. There are different varieties of encapsulation procedures, such are physical processes (air suspension coating, coextrusion with submerged or stationary nozzle, rotating disk atomization, spray drying or spray cooling), but also a variety of chemical encapsulation processes (matrix polymerization, liposome technology, simple or complex coacervation, solvent extraction, evaporation of solvents and others. Encapsulation by coacervation is a technique that involves production of core–shell microcapsules by using the interaction of oppositely charged polyelectrolytes in aqueous form. It is characterized by high payload and high encapsulation efficiency (higher than 99%), relatively low costs, and ability to use ambient room temperature and pressures [24]. Coacervation is a process in which an electrostatic interaction between two aqueous media occurs. There are many different types of hydrophilic polymers such as sodium alginate, gelatin, and chitosan that are usually used for the creation of polymeric micro-or nano-capsules [25].

Coacervation includes phase-separation for creation of two separated liquid phases. The first of them consists of a polymer-rich phase (coacervate) and the second one of polymer-depleted phase (equilibrium solution). Therefore, the process of coacervation can be used for both polar and non-polar species. In the case of more complex coacervation systems, an active ingredient can be encapsulated by the combination of two species, such as gelatin and gum arabic or sodium alginate and chitosan [26]. Chitosan is insoluble in alkaline pH medium, but it precipitates in contact with an alkaline solution. During this reaction, the particles are produced by adding the chitosan into an alkali solution using a compressed air nozzle. Lastly, the separation of encapsulated particles is achieved by filtration [27]. 

Coacervation is one of the oldest and most widely used techniques of microencapsulation. Based on the process, it can be divided into simple coacervation (using colloidal gelatin or chitosan, and complex coacervation in which the polymeric solution is prepared from two oppositely charged colloids, such as gelatin with gum arabic or chitosan (Table 1) [28].

After creating antimicrobial microcapsules filled with silver active substances, such microcapsules need to be fixed at the surface of the polymer materials [28,29,30,31]. Dip-coating immersion is a process that is ideally fitted for such purpose since in it the substrate is immersed in a liquid and then extracted at a certain speed at a controlled temperature and atmospheric conditions. Antimicrobial components are present in the liquid form [32,33,34]. Recent focus is on the application of gold or silver nanoparticles [35,36,37] and for the characterization of such materials particular methodology and combination of spectroscopic and microscopic techniques is needed [38,39,40]. The process has three stages, which include immersion of the substrate, the forming of a wet layer and drying of the coating by solvent evaporation. Immersion is very slow, allowing the coating to be oriented into a more favorable, denser structure. Evaporation of the solvent can cause destabilization if it is not performed under favorable conditions. During this process the cross-linking occurs, in which the coating becomes stiffer and the resulting gel is glued by heat, while the coagulation temperature itself depends on the composition [41,42,43]. Many different parameters influencing the outcome and the thickness of the film during immersion exist; these are the viscosity of salts, surface tension, and pressure. By optimizing these parameters, the efficient adhesion of microcapsules is fixed to the surface of the polymer. Moreover, the antimicrobial effects are enhanced with silver nanoparticles [44,45,46]. Colloidal nanoparticles have many benefits [47,48,49,50], and their characterization is achieved with sophisticated methodology [51,52].

The antimicrobial core of microcapsules contains silver. Interesting work from Pandey et al. [6] has shown that the method of stabilization of silver nanoparticles strongly influences antimicrobial properties. In their research, the silver nanoparticles (AgNPs) were even more toxic to bacteria than to other microorganisms like fungi [6]. Thus, the stabilization of silver nanoparticles has broad antibacterial applications [7,8,9,10,11]. Encapsulated active species need to be glued to the surface of the polymers, and this is achieved by dip-coating methodology which uses the sol–gel mechanism of binding microcapsules to the textile carrier [53,54,55,56].

The sol-gel is one of the oldest procedures used on thin films. It results in a uniform coating that is very suitable for antimicrobial protection [57]. Due to their properties, microcapsules can be used in various branches of industry [58]. It is therefore not surprising that several studies are being conducted aimed at developing formulations intended for healthcare, especially as an integral part of textiles [59,60]. This work was focused on the functionalization of polymer surfaces by microcapsules filled with antimicrobial silver. The novelty and significance of this work included functionalization of the polymer surface not directly with nanoparticles, as is conventionally performed, but with shell/core microcapsules filled with antimicrobial silver compounds that enable release of antimicrobial silver in time and by this enhances the efficiency of the antimicrobial properties of the functionalized polymer. More precisely, this work is in optimization of the microencapsulation process through two steps of simple and complex coacervation for further effective functionalization of polymers by dip-coating methodology.

The main hypotheses of our work are the following: (i) antimicrobial microcapsules can be prepared with antimicrobial silver in different sizes and chemical compositions; (ii) the microcapsules can be easily applied on the surface of the polymers by dip-coating methodology; and (iii) novel materials with microcapsules release Ag in time, so they can be used as medical materials.

## 2. Materials and Methods

### 2.1. Materials and Instrumentation

In this investigation, the following reagents of highest purity were used: the dithizone, 1,5-diphenylthiocarbazone (C_13_H_12_N_4_S) manufactured by Merck (Darmstadt, Germany), Ethanol 96% (C_2_H_5_OH) manufactured by Gram-Mol (Zagreb, Croatia), silver nitrate (AgNO_3_) manufactured by Gram-Mol (Croatia), zinc sulfate 7-hydrate (ZnSO_4_ × 7H_2_O) manufacturer Gram-Mol (Zagreb, Croatia), 3-glycidyloxypropyltrimethoxysilane (GLYMO, 98%) manufactured by Aldrich Chemicals, sodium alginate (C_6_H_9_NaO_7_) manufacturer Sigma-Aldrich for testing.

Buchi Encapsulator B-390 manufacturer Buchi Labortechnik AG (Flawil, Switzerland) was used for encapsulation. Heidolph Unimax 1010, manufactured by Heidolph Instruments (Schwabach, Germany), was used to mix the aqueous medium with the microcapsules. A two-beam UV/VIS Lambda 20 spectrometer manufactured by Perkin Elmer (Waltham, MA, USA) was used to perform spectroscopic analysis. During the measurements, 10 mm quartz cuvettes manufactured by Perkin Elmer (Waltham, MA, USA) were used. 

The samples after microcapsulation were tested using high-resolution optical microscopy (Tescan Vega company, Brno, Czech Republic) and afterwards a scanning electronic microscope “TESCAN VEGA TS5136LS” with an EDS detector. A microphotograph obtained from SEM microphotograph of encapsulated antimicrobial silver, as well as EXD analysis of the samples core and shell parts, are presented in the Results section.

### 2.2. Microencapsulation of Antimicrobial Silver

**Figure 1:**
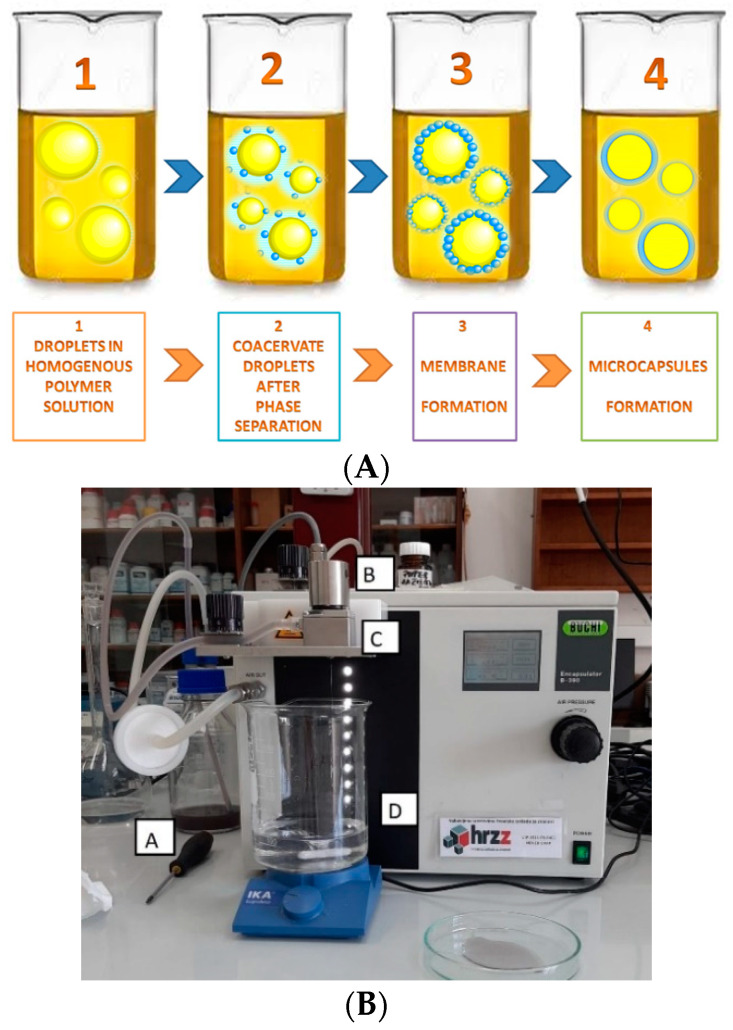
(**A**) Steps in encapsulation by coacervation prior the functionalization, (**B**) encapsulation apparature Buchi Encapsulator B-390 manufacturer Buchi Labortechnik AG (Flawil, Switzerland) (A—500 mL bottle with encapsulating agent; B—magnetic vibrator; C—heater; nozzle at the bottom; D—solidifying agent).

In this work, the encapsulation of the silver-filled microcapsule samples was performed by simple coacervation, within the sodium alginate samples that contained zinc sulfate 7-hydrate in its outer shell. Microcapsulation is a process that involves the separation of a macromolecular solution into two immiscible liquid phases: the first is a denser coacervate phase and the second one is a diluted equilibrium solution. Four basic steps are carried out under stirring and are presented in Figure 1 including: (1) dispersion of the active substance into a solution of a surface-active colloid; (2) precipitation of the colloid onto the dispersed droplets; (3) addition of a second colloid to induce the polymer–polymer complex coacervation; and (4) stabilization by adding a cross-linking agent chitosan (Figure 1) [28].

In this work, the encapsulation of the silver-filled microcapsule samples was performed by reaction in which polymer anions react with polyvalent cations, whereupon an outer shell is formed. The process is most commonly done with the aid of external ionotropic method who’s steps are presented in Figure 1, including: (1) polymer droplets containing an active ingredient are immersed in a homogenous solution containing the crosslinker; (2) crosslinker interacts with the polymer; (3) formation of the outer shell [28]. Encapsulation was performed under the following conditions: pressure 530 mbar, amplitude 5, frequency 2000 Hz and heating 50 °C. During the encapsulation, the occurring droplets of the solution were falling into the zinc sulfate 7-hydrate solution. After completion of encapsulation, the resulting microcapsules were stirred for 60 min, filtered and washed with distilled water. The resulting microcapsules had a core filled with silver ions, and a shell composed of sodium alginate and zinc sulfate 7-hydrate. The encapsulation procedure was performed using a Buchi Encapsulator B-390. To perform the encapsulation, it was first necessary to prepare a solution of sodium alginate with a concentration of 1.5%, and a solution of zinc sulfate 7-hydrate with a concentration of 1M. A 1.5% sodium alginate solution was prepared by dissolving 7.50 g of sodium alginate in a 500 mL volumetric flask with distilled water.

Zinc sulphate 7-hydrate, on the other hand, was prepared by weighing 143.77 g of zinc sulphate 7-hydrate into a 500 mL volumetric flask, then adding water to the mark after which the contents of the volumetric flask had to be shaken well. After the sodium alginate was dissolved, a solution of sodium alginate and AgNO_3_ was prepared. For this purpose, AgNO_3_ weighing 0.0332 g was dissolved in a solution of sodium alginate with a volume of 100 mL, with stirring on a magnetic stirrer. The dissolution process was performed for 30 min. After dissolution, the solution had to be filtered using vacuum filtration. Prior to the encapsulation procedure, 100 mL of zinc sulfate 7-hydrate solution was poured into a 600 mL beaker. Zinc sulfate 7-hydrate served as a solidifying agent in the encapsulation process. In a 500 mL bottle, 100 mL of the filtered solution was poured, which, thanks to nitrogen pressure, passed through a silicone tube all the way to a 20 μm diameter nozzle. When passing all the way to the nozzle, a silicone tube passes by a heater that heats the solution, thus preventing the solution from clotting in the nozzle. At the same time, a magnetic vibrator was used to ensure the formation of as many microcapsules as possible. The encapsulator with the indicated parts is shown in Figure 1.

During encapsulation, droplets of the solution fall into the solidification solution, i.e., zinc sulfate 7-hydrate solution. After completion of encapsulation, the resulting microcapsules were stirred for 60 min to ensure solidification. After solidification, the microcapsules were filtered and washed with distilled water. The resulting microcapsules had a core composed of AgNO_3_, and a shell with zinc sulfate 7-hydrate. The microcapsules were purple, but staining caused by the presence of AgNO_3_ occurred.

After 30 min, the spheres were filtered, rinsed and stored in a cool and dark place prior to the characterization. Part of the microcapsules was dispersed in chitosan solution (0.5% CS in 1.0% CH_3_COOH) under constant stirring (magnetic stirrer). The contact time between microspheres and chitosan solution was about 30 min to give chitosan time to form a layer around the microspheres and formation of microcapsules. Microcapsules were filtered, rinsed and stored in a cool and dark place prior to the characterization. A number of the microcapsules were allowed to air-dry at room temperature to reach their equilibrium moisture content.

### 2.3. Monitoring of the Release of Silver Ions

The active substance is released from the microcapsule by diffusion, rupture or dissolution of the coating. In this case the active substance, Ag^+^ cations, is released by diffusion. To perform diffusion, it was first necessary to weigh, into a 100 mL beaker, 1 ± 0.0001 g of microcapsules. Random sampling was used to select microcapsules. After the weighing, 20 mL of distilled water was added via a pipette. A total of 23 aqueous solutions prepared in this way were used for testing. Afterwards, the solutions were shaken. The Unimax 1010 (manufacturer Heidolph instruments, Schwabach Germany) instrument was used for shaking. The shaking conditions were: default shaking speed 4 (the instrument has 10 possible speeds); shaking time 60 min; no heating. After 60 min, the solutions were filtered to separate the microcapsules, as microcapsules can disrupt spectrometric analysis.

### 2.4. UV-VIS Characterization

To monitor the course of the analysis, and to determine the concentration of Ag^+^ cation, it is necessary to make a calibration diagram, i.e., the calibration curve. To perform quantitative and qualitative analysis, a two-beam UV/VIS Lambda 20 spectrometer was used. The test was performed in the UV and visible range, i.e., in the wavelength range 190 to 900 nm. Since the test was performed in the UV range, cuvettes made of quartz were used.

Before the test, the dithizone solution had to be diluted 1:9 with distilled water in a 10 mL volumetric flask. At the same time, the microcapsules contain zinc sulfate 7-hydrate, so it is possible that Zn interferes with the determination of silver. In order to reduce interference, i.e., to better detect the signal from the Ag^+^ cation, the standard addition method was used. The standard addition method involves adding a standard solution of known concentration to a volumetric flask containing the test solution. In order to perform the UV-VIS spectroscopical investigation of colorless silver solution, first it was necessary to create a colored complex with dithizone (diphenylthicocarbazone), Sodium diethyldithiocarbamate, 4-(2-Pyridylazo) resorcinol monosodium salt, and Methyl N-[α-(8-hydroxy-7-quinolyl) benzyl] anthranilate. Dithizone, a complex compound that can be dissolved in carbon tetrachloride, chloroform, and 96% ethanol, was used for testing [61]. To avoid the use of toxic chemicals such as carbon tetrachloride and chloroform [62,63], 0.07% dithizone solution was used for testing purposes, which had to be filtered before use to remove residual dithizone residues. In the analysis, it is important to keep in mind that dithizone itself is not selective and reacts with all metals present in the test solution [63]. Before performing the analysis, it was necessary to make a calibration diagram. For the purpose of making the calibration diagram, a titron of AgNO3 with a concentration of 0.1 M was used as a standard solution. The calibration diagram was made in the following concentrations of standard solution c_1_ = 4.444 × 10^−3^ M; c_2_ = 8.888 × 10^−3^ M; c_3_ = 0.0178 M; c_4_ = 0.0222 M; c_5_ = 0.0444 M. Standard solutions were added with dithizone to 25 mL volumetric flasks in a 1:1 volume ratio, achieving an excellent correlation of the calculated absorbances.

### 2.5. Chromatographic Characterization

The TLC method was developed for the analysis of metal ions that can be extracted from the microcapsules. During preliminary experiments, more than 30 stationary phases were investigated with different mobile phases. Hydrochloric acid and water were added to create optimal solvent mixtures. The chromatographic method used contained ACN, HCl, and H_2_O in 60.00:19.17:20.83 *v*/*v*%, respectively.

### 2.6. Dip Coating of Polymers with Antimicrobial Microcapsules

The dip-coating process was performed in a following manner: firstly the sol was stirred magnetically to obtain optimal molar ratio of GLYMO to water, based on our previous experience to retain textile character (hand value, softness, etc.) [42,47,55]. Important parameters for the sol–gel properties (precursor, types of catalysts and solvent, molar ratio of precursor to solvent, pH value, and temperature) were also based on physical and chemical character of the chosen precursor GLYMO and catalyst, HCl.

Secondly, the solution of distilled water and ethanol was thoroughly mixed, since volatile substances can be removed from sol by evaporation before or during condensation. Then, GLYMO was added and the process was carried out under continuous magnetic stirring until a homogeneous solution was obtained. Thirdly, the samples were cut as to be in 5 × 5 cm^2^ in size in order to go through the dip-coating process on a custom-made apparatus with the predetermined drawing speed of 1 mm/s. Lastly, such modified samples were left to gel at room temperature for 24 h, and were then dried at 100 °C for 1 h. For the antimicrobial treatment effects, silver ions originating from silver nitrate were added to the sol carefully and in small amounts, under higher mixing speed. The process was carried out under continuous magnetic stirring until a homogeneous solution was obtained.

The detailed description of the surface modification by using the sol-gel procedure was optimized in our previous work [47] where we introduced the technology of polymer functionalization by 3-glycidyloxypropyltrimethoxy-silane (GLYMO, Sigma Aldrich, Darmstadt, Germany, Europe). Nanoparticles in microcapsules were left to gel at room temperature and then heated at 100 °C for 60 min. Such modified products were characterized by different instrumental techniques, including thin layer chromatography (TLC), scanning electron microscopy with an EDX detector (SEM-EDX, Tescan Vega, Brno, Czech Republic), and FTIR-ATR (Perkin Elmer, Waltham, MA, USA) spectroscopy [47]. In this work, the results of modification by microcapsules filled with silver compounds will be shown. There are many crucial factors that influence the efficiency of the functionalization process: the speed of immersion, concentration and related viscosity of solutions, the surface tension, and the sample dimensions [43]. Dip coating was applied on both woven and nonwoven polymers. The important working parameter was the molar ratio of GLYMO to water, which was chosen based on the experimental results obtained. We observed that 1:1.5 is the stoichiometric ratio for total condensation of the alkoxide groups and 1:3 is the stoichiometric ratio for total hydrolysis of the alkoxide groups. The optimal molar ration was chosen from the obtained resulting surface: for example, the goal to sustain the homogenous distribution of antibacterial silver on the coating, smooth touch, optimal shine and the most flexible surface coating that resists force without cracking, was associated with optimized system parameters.

The last working parameter that influenced the dip-coating process was the composition of the sample. It was determined in accordance with the standard norm procedure ISO 1833.22. The sample was composed of cellulose fibers. Mass per unit was determined in accordance with the ISO 2286-2 and found to be 186.7 g m^2^. No preparatory process on the textile substrate before dip-coating was performed (scouring, bleaching, or others).

### 2.7. FTIR Spectroscopical Characterization

FTIR characterization of polymers functionalized with antimicrobial microcapsules, was done by Fourier transform infrared spectrometer (Spectrum 100 FTIR, Perkin Elmer, Waltham, MA, USA), which applies KBr and Attenuated Total Reflectance (ATR) techniques. The spectra of samples were recorded in a frequency range from 400 to 4000 cm^−1^ in diffuse reflectance mode, at a resolution of 4 cm^−1^. This instrument with this particular configuration enables the recording of spectra of the sample solid-state.

## 3. Results and Discussion

The results of this investigation are a step forward in finding the solutions against powerful resistant microorganisms. Microencapsulation is a fascinating technique that enables slow release of antimicrobial agents, can be applied on different materials without affecting the existing polymer, and has a valid shelf life. The focus of this investigation was on antibacterial silver ion that was the core compound of all prepared microcapsules. This material was chosen since it is known for its strong antimicrobial effects against a wide range of Gram-positive and Gram-negative bacteria, including antibiotic-resistant strains. Encapsulation methods enhance the applications of antimicrobial silver. The availability of microcapsules to slowly release silver has enabled potential storage, stabilization, and application in many different products. In this work, microcapsules were prepared as antimicrobial materials for medical applications (for example, on bandages, wound materials, sheets in hospitals, and others. The antimicrobial silver was chosen due to the reported literature references, and based on our testing. We reported the results of the antimicrobial investigation of the silver nanoparticles in our previous investigations [42,47], in which we determined the antimicrobial activity on *S. aureus* model microorganisms.

### 3.1. Characterization of Microcapsules with Antimicrobial Silver

**Figure 2:**
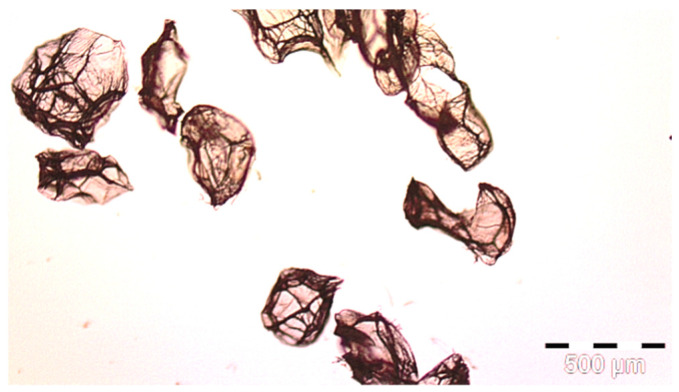
Microphotograph of prepared microcapsules filled with silver ion of 450 μm in diameter, prior functionalization of polymers recorded by optical microscopy with high resolution.

Figure 2 shows the microcapsules prepared by simple coacervation process, filled with silver ions in the core, and the alginate shell with zinc sulfate 7 hydrate in the shell.

Some microcapsules were also encapsulated by additional adding of chitosan, during complete coacervation process. No other physical, chemical or morphological property of the antimicrobial silver filling of the microcapsules was varied during their production. Particularly, the focus was not to change any step of modification and functionalization on the polymer surface. To conclude, there was no difference among the final filling product of microcapsules.

**Figure 3:**
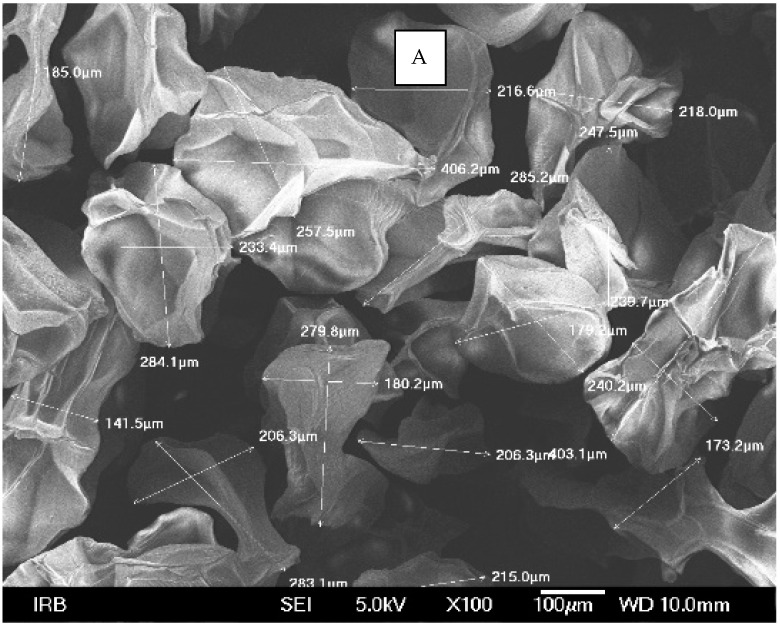

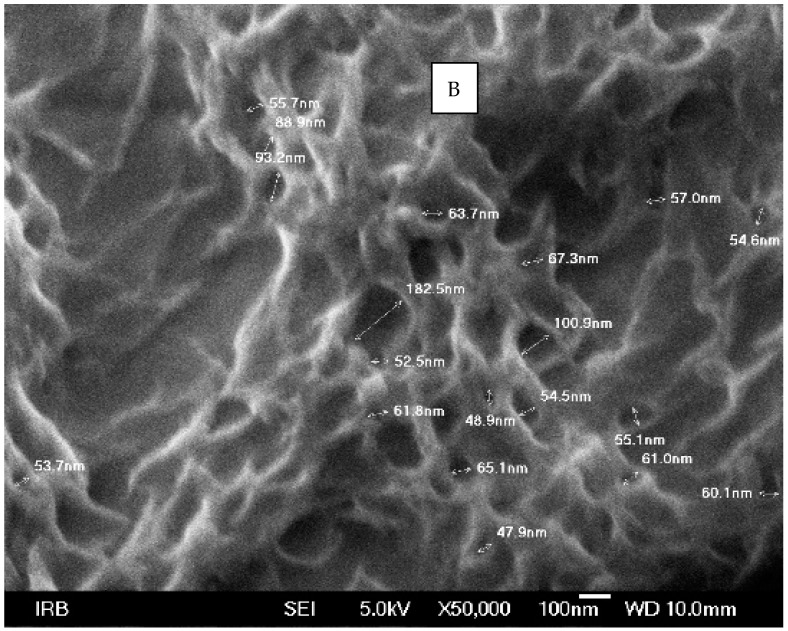
SEM microphotograph of prepared microcapsules filled with silver ions: (**A**) determination of dimensions of the liophilizated microcapsules and (**B**) calculated dimensions of the pores on their surface.

After recording microphotographs of microcapsules after encapsulation using high-resolution optical microscopy, a scanning electron microscope was used in order to determine the dimensions of pores and shells of particular microcapsules after their lyophilisation. The combination of both techniques has been widely used for the characterization of microcapsules. The SEM results are presented in Figure 3A. As can be seen from this Figure, the average diameter of microcapsules was in the range of 140 to 285 μm in diameter. When comparing those results to the results presented in Figure 2, it can be concluded that the reduction in size of microcapsules after their lyophilization was in range of 37% to 69%. The most important result is the proof that there were no cracks on microcapsules after the lyophilization process. This points to the conclusion that the lyophilization process was adequate, not causing any damage to the shell of microcapsules. Similar results were obtained and discussed by Glaucia et al., 2013 [56], who discussed that these characteristics are important to ensure greater protection and retention of the encapsulated material. In their research the encapsulated sucralose by double emulsion followed by complex coacervation was cross-linked by solid bridges.

The results followed to conclusion that such solid bridges can be attributed to the lyophilization process, which was responsible for clustering of the microcapsules. [56]. Moreover, their results are in agreement to ours in which it was shown that the average particle size was not influenced by varying the shell material. However, Glaucia et al. observed that the concentration of encapsulating agent has influence on the average particle size [56]. In contrast, He et al. [57] did not use lyophilization but spray drying of the microcapsules prior to the SEM investigation.

Their results showed that the particle sizes of spray-dried microcapsules were distributed in much broader range, between 1 and 100 μm, while the most of them were distributed around 10–30 μm [57]. As can be seen from Figure 3A, in which the results of our research obtained by SEM are shown, the determined size of microcapsules was in range of 150 to 400 nm, while the size of the pores among them microcapsules was in range of 50 to 100 nm.

**Figure 4:**
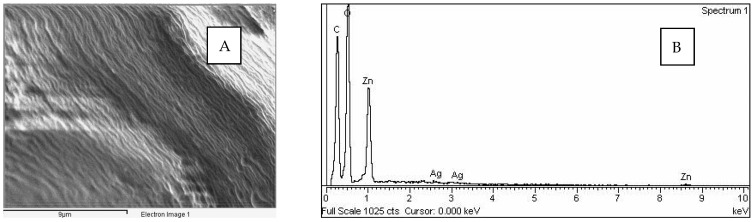
SEM-EDX chemical characterization: (**A**) the surface of the sample that was used for characterization; (**B**) SEM-EDX spectra showing the Ag in core and Zn in shell of antimicrobial microcapsules.

The results of SEM-EDX presented in Figure 4 show that the microcapsules contain zinc and silver. In order to investigate the release of silver from the core of the microcapsules, further investigation by chromatographical screening and quantitative UV-VIS spectroscopy was performed.

### 3.2. The Results of Chromatographic Prescreening

Silver from microcapsules were qualitatively identified by using thin-layer chromatography. The chromatographic investigation enables monitoring of silver among other metals, due to its distinguished R_F_ value. In the chromatographic system that has ACN: HCl: H_2_O in 60.00: 19.17: 20.83 *v*/*v*%, respectively, silver was detected by different indicators (Table 2).

### 3.3. The Results of UV-VIS Characterization

**Figure 5:**
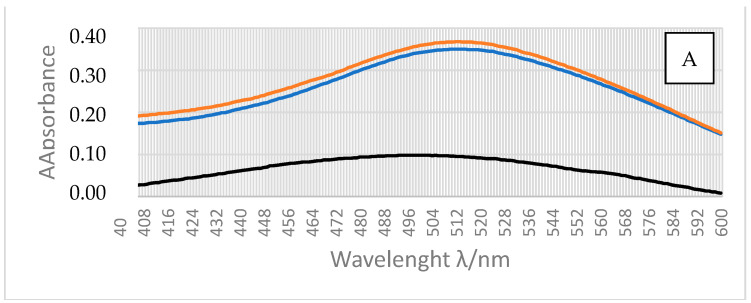

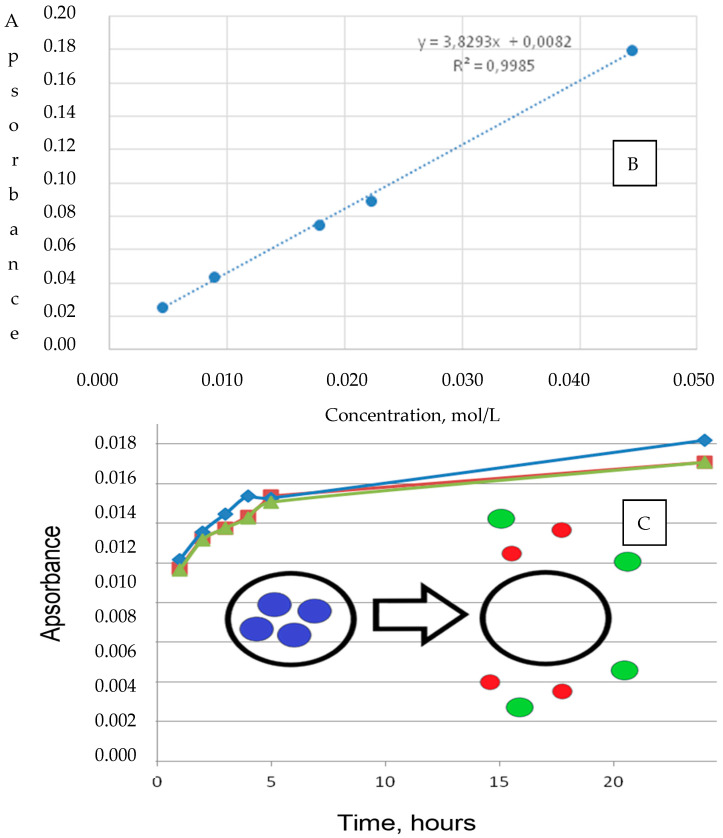
(**A**) UV-VIS curves obtained during measurements (black—silver from microcapsules; blue—standard reference solution with silver ions; and orange—mixture of silver from microcapsules and standard solution, spike solution); (**B**) calibration diagram of silver ion obtained by UV-VIS spectrometry; and (**C**) graphic representation of the silver release over 25 h from microcapsules (blue—450 μm microcapsules without chitosan, red—120 μm microcapsules without chitosan, green 120 μm microcapsules with chitosan). As can be seen in details, the release starts in the first 4 h and then it reaches a meta-stabile phase.

The UV-VIS characterization was performed in a range of 400 to 600 nm. Figure 5A presents the UV-VIS curves obtained during investigation, and Figure 5B the calibration curve calculated based on the results. In order to make the calibration curve, five AgNO_3_ solutions of known concentrations were used. All samples were prepared in the same manner: firstly 1 mL of dithizone was added to five 25 mL volumetric flasks with AgNO_3_ solutions. Secondly, the same volume of dithizone as AgNO_3_ solutions was added to the samples. All absorption values were recorded at a maximal wavelength of 512 nm. The values of the recorded absorptions are shown in Figure 5A,B.

Before the analysis of the release of silver from antimicrobial microcapsules, the samples had to be determined. The calibration curve presented in Figure 5B was used for the determination of release of the silver (Figure 5C). During the diffusion in an aqueous medium, Ag^+^ cations “exit” from the microcapsule into the medium itself. The diffusion procedure itself is shown in Figure 5C, together with the obtained spectral curves. Quantitative analysis of silver in microcapsules of different diameters (120 or 450 μm) showed that samples contained between 100 and 350 μg/g of silver, respectively. Those amounts are valid for protective medical materials.

### 3.4. The Results of Functionalization of Woven and Non-Woven Polymers by Microcapsules

Table 3 presents the microphotograph of the samples after dip-coating functionalization recorded under the optical microscopy with specified chemical composition (woven and non-woven cotton and viscose polymers).

### 3.5. The Results of Spectroscopical and Microscopical Characterization

**Figure 6:**
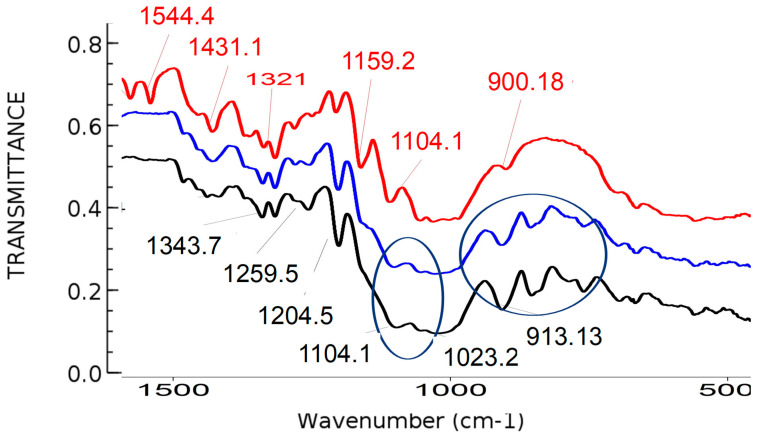
Fourier-transform infrared spectra of the samples before and after the modification: red line—FTIR-Attenuated Total Reflectance (ATR) spectra of cotton woven polymer sample before modification with GLYMO precursor; blue line—FTIR ATR spectra of cellulose sample after modification with organic GLYMO precursor; black line—FTIR ATR spectra of cellulose sample modified with GLYMO precursor and silver. Two blue circles define area of the epoxy groups determined around ~905 and 911 cm^−1^, and the area around 1100 cm^−1^ is linked to Si–O groups.

For the spectroscopic characterization of the samples, the Fourier-transform infrared spectrometry (Spectrum 100 FTIR, Perkin Elmer, Waltham, Massachusetts) was used, and the result is shown in Figure 6. The newly obtained microcapsules were then impregnated by dip-coating process on 100% viscose nonwoven fabric, and woven viscose or viscose/cellulose polymers. As can be seen from Figure 6, the dip coating process can be monitored through the formation of particular functional groups that are present after the formation in a sol-gel process.

ATR-FTIR is a spectroscopic methodology that allows analysis of the microcapsules without complicated sample preparation which can significantly increase the speed of analysis and the number of sample throughput. In determination of the shell thickness of antimicrobial microcapsules, the ATR-FTIR technique has a capability to penetrate in the micrometer range. Therefore, it is generally used to provide information on the functional groups, characterizing the microcapsules’ shell properties.

Microcapsules were prepared in this research as a career of antimicrobial agents, particularly antimicrobial silver. In our previous research we have investigated the effects of different metal and metal-oxide nanoparticles, and our results have shown that small metal silver nanoparticles have very strong antimicrobial effects, not only against conventional Gram positive and Gram negative bacteria, but also against fungi. In addition, their application on the surface of polymers that were used in this research have resulted in activity against drug resistant methyl resistant staphylococcus aureus MRSA and methyl sensitive staphylococcus aureus MSSA strains.

In this work, the antibacterial effect of our antimicrobial polymer coated with microcapsules was based on previous research on antimicrobial effects of silver that started after 6h of exposure [42,47]. Therefore, the concentrations used for the research were chosen based on the parameters of the maximal antimicrobial efficiency of silver. The application of antimicrobial silver as potential material for bandages and open wounds was the main focus of this work (Table 1). 

The potential strong efficiency of the newly formed materials is a result of functionalization that is shown in figures and tables, but particularly, at the FTIR-ATR spectra of samples after modification. Therefore, the antibacterial microcapsules were efficiently applied to the polymer surface and were filled with antimicrobial silver.

### 3.6. The Results of Antimicrobial Testing

The results of this investigation have shown that such surfaces might possess an antimicrobial effect due to the presence of silver particles, but this is a matter for future studies.

In our previous papers [42,47] we have reported that silver is a potential strong antimicrobial reagent. Its efficiency is enhanced when it is present in a form of nanoparticles. Such samples in size of 10 nm showed even potential antimicrobial investigation of metal and metal oxide nanoparticles on model microorganism *Staphylococcus* *aureus* ATCC 29213 by agar-well diffusion assay and reported zone of inhibition was 10 mm for 20 ppm of silver.

In their work, Özyildiz and co-workers [53] sought to develop antimicrobial textiles by using encapsulated ozonated red pepper seed oil in a wrapper made of gelatin and Arabic rubber. Our results correlate with the literature data [53], where the authors concluded that the processed textile materials show good antimicrobial properties against the bacteria *Staphylococcus aureus, Candida albicans*, and *Escherichia coli* [53]. In order to have enough data on comparison of microcapsules made by two different approaches—simple and complex coacervation—much more investigation needs to be performed, and this will be the focus of our further research. We have reported the results of our previous work, although there are many different sources that specify different antimicrobial activity based on the chemical and physical characteristics of particular nanoparticles. As is emphasized in this work, much more data should be collected in future work in order to clearly specify that new material is antimicrobial efficient, but for work focused on the preparation and fixation of newly prepared microcapsules on woven and non-woven materials.

In contrast to our research, Ramya and Maheshwari [54] applied microcapsules with a piper betel leaf extracts encapsulated inside the microcapsules. Unlike Özyildiz et al., they used fabrics composed of 50:50% (*W*/*W*) of cotton and bamboo blend fibers. At the same time, they used the method of exhaustion to apply microcapsules to textile material. Moreover, for the encapsulation purposes, the authors used spray encapsulation in which calcium chloride was sprayed on the drops of extract and sodium alginate. The authors stated that their result was a textile material with antimicrobial properties effective against bacterial species that are often found on foot wounds: *Staphylococcus, Bacillus, Klebsiella, Pseudomonas*, and *Proteus*. For this purpose, the antimicrobial testing was performed on materials containing piper betel extract, and on materials containing microcapsules with the encapsulated extract. Materials treated with microcapsules showed better results, especially after washing [54].

The antibacterial activity and mechanism of silver on *S. aureus* strain ATCC 6538P were investigated by Li and coauthors [44]. Their results have shown that the minimal bactericidal concentration (MBC) is 20 μg/mL. Those results can be compared to the results of other researchers; such is the paper by Mirzajani and his coworkers [45]. In this paper the Mirzajani et al. showed that 4 μg/mL of silver completely inhibits bacterial growth. This interaction was confirmed by Grigoreva et al. [46]. Shevtsova et al. [58] incorporated antimicrobial silver nanoparticles inside temperature-responsive hybrid nanomaterials based on modified halloysite nanotubes, an approach that offers many significant improvements to the application of antimicrobial materials. Moreover, Raczkowska et al. [59] presented results of “smart” antibacterial surfaces based on silver nanoparticles (AgNPs) embedded in temperature-responsive poly(di(ethylene glycol)methyl ether methacrylate)—(POEGMA188) as well as poly(4-vinylpyridine)—(P4VP) coatings attached to a glass surface. They managed to provoke a temperature-switched killing of the bacteria against *Escherichia coli* ATCC 25922 and *Staphylococcus aureus* ATCC 25923 [59]. Konnova et al. reported a simple procedure for fabrication of 50 nm silver nanoparticles and application of these nanoparticles against yeast or bacteria. They have applied cationic polymer-stabilized nanoparticles that were electrostatically adhered to microbial cells. By such forms, they were producing an even monolayer on the cell walls that resulted in fast delivery of silver nanoparticles into *C. elegans* micro worms. The fabrication of “cyborg” biological cells that contain surfaces functionalized with nanomaterials is today a fascinating new area in cell-surface engineering that is promising for many new applications. Therefore, it can be concluded that the results of our research correspond to the relevant and recent literature data. Moreover, we have shown that silver is one of the most prominent candidates for application on antibacterial medical materials produced by encapsulation.

## 4. Conclusions

Due to the growing resistance of harmful bacteria to conventional drugs, it is necessary to develop new solutions and new formulations intended to suppress their growth on medical textile materials. Encapsulation was proposed in this research as a fascinating technique that enables effective new ways of partial release of antimicrobial agents. We produced microcapsules of 120 and 450 μm from sodium alginate and zinc sulfate 7-hydrate, filled with silver, and characterized them by different microscopic and spectrometric methodologies. Afterwards the microcapsules were efficiently bonded to the surface of woven and nonwoven polymers (made of viscose and cellulose), for preparation of antimicrobial materials. Lastly, the release of active antimicrobial silver from microcapsules was monitored over 25 h in an aqueous medium in order to predict the antimicrobial potential of microcapsules, as potential filler for medical materials such are bandages and wound dressings. By this, it can be concluded that the main hypothesis of this work were proven: (i) antimicrobial microcapsules were efficiently prepared with antimicrobial silver in different sizes (120 and 450 μm) and chemical compositions (with and without chitosan) (ii) the microcapsules were efficiently applied on the surface of the polymers by dip-coating methodology; and (iii) novel materials with microcapsules released Ag in time so this is the proof that such items can be used as medical materials. Finally, based on the obtained results, the antimicrobial efficiency can be predicted to inhibit the growth of certain strains of microorganisms on open wounds.

## Figures and Tables

**Table 1:** Shell and core materials used in simple and complex coacervation for textile applications.

Simple Coacervation		
Polymer	Coacervation Agent	Active Substance
Chitosan	NaOH	Antimicrobial silver, essential oil
Chitosan	Sodium dodecyl sulphate	Antimicrobial silver, linseed oil
Chitosan	Sodium tripolyphosphate	Antimicrobial silver, honey, vitamins
**Complex coacervation**		
**A Polymer**	**B Polymer**	**Active substance**
Gum arabic	Gelatin	Antimicrobial silver ions
Gum arabic	Chitosan	Antimicrobial silver, oils, perfumes, dyes
Chitosan	Gelatin	Antibacterial silver, essential oils

**Table 2:** Thin-layer chromatography results of detected silver after using different visualization reagents; development by ACN: HCl:H_2_O in 60.00:19.17:20.83 *v*/*v*%, on cellulosic precoated plates 20 × 20 cm.

Detection of Silver through Its R_F_ Value and Characteristical Colors of Spots			
Without Indicator	Dimethylglioxime	Quercetin	Alizarin Red S
+	+	+	+ Zn can be detected as pink spot

**Table 3:** Microphotograph of the samples after dip-coating functionalization (woven and non-woven cotton and viscose polymers).

	Before Modification, without Microcapsule	After Modification with Yellow Microcapsules
**Woven and cotton**	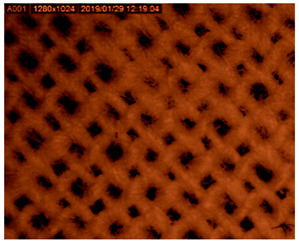	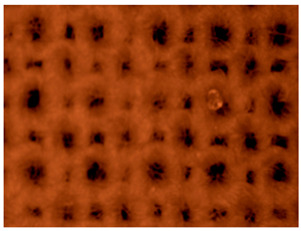
**Woven cotton**	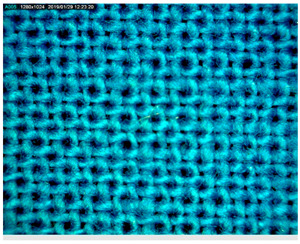	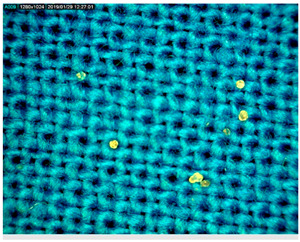
**Non woven, viscose**	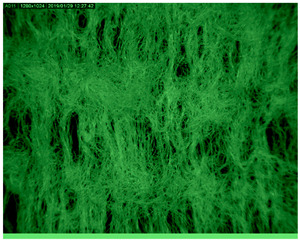	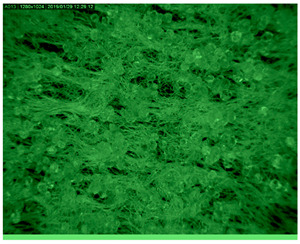

## Data Availability

Not applicable.
